# Synchronous bilateral renal cell carcinomas with differing histologies

**DOI:** 10.1002/iju5.12186

**Published:** 2020-06-28

**Authors:** Ryu Shigehisa, Takashi Karashima, Shu Kobayashi, Kaya Atagi, Daigo Takemori, Hideo Fukuhara, Satoshi Fukata, Ichiro Murakami, Naoto Kuroda, Keiji Inoue

**Affiliations:** ^1^ Department of Urology Kochi Medical School Nankoku Japan; ^2^ Laboratory of Diagnostic Pathology Kochi Medical School Hospital Nankoku Japan; ^3^ Department of Diagnostic Pathology Kochi Red Cross Hospital Kochi Japan

**Keywords:** bilateral, clear cell renal cell carcinoma, mucinous tubular and spindle cell carcinoma, renal cell carcinoma, synchronous

## Abstract

**Introduction:**

Bilateral renal cell carcinomas with different histological types are rare. We report herein the first description of bilateral renal carcinomas with clear cell renal cell carcinoma and mucinous tubular and spindle cell carcinoma occurring synchronously.

**Case presentation:**

A 62‐year‐old man was referred to our hospital with bilateral renal tumors. The tumors on each side showed different findings from both contrast‐enhanced computed tomography and magnetic resonance imaging. The tumors were partially resected. Histopathological and immunohistochemical examination of the left renal tumor diagnosed clear cell renal carcinoma. Histopathological and immunohistochemical examination of the right renal tumor diagnosed mucinous tubular and spindle cell carcinoma.

**Conclusion:**

We encountered a case with clear cell renal cell carcinoma and mucinous tubular and spindle cell carcinoma occurring simultaneously in bilateral kidneys.

**Abbreviations & Acronyms**
AMACR: α‐methylacyl‐CoA racemase
CA9: carbonic anhydrase 9
ccRCC: clear cell renal cell carcinoma
CD10: cluster of differentiation 10
CK7: cytokeratin 7
CT: computed tomography
DWI: diffusion‐weighted imaging
HE: hematoxylin and eosin
MRI: magnetic resonance imaging
MTSCC: mucinous tubular and spindle cell carcinoma
RCC: renal cell carcinoma

**Keynote message**

Bilateral RCCs with differing histologies are rare. This is the first report of ccRCC and MTSCC occurring synchronously. Diagnostic imaging is crucial to prioritize therapy.

## Introduction

The incidence of bilateral RCCs diagnosed synchronously is 1–5%.^1^, ^2^ We report herein the first description of a patient with ccRCC and MTSCC occurring synchronously in bilateral kidneys.

## Case presentation

**Fig. 1:**
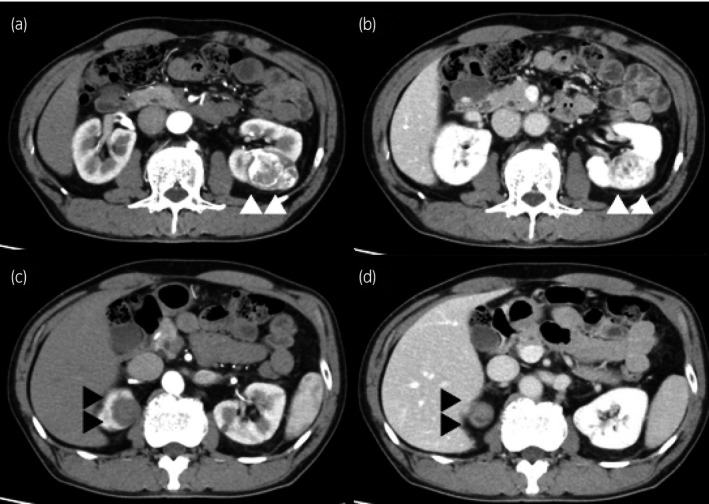
Preoperative CT of bilateral renal tumors. The left renal tumor is 42 mm in diameter and shows well‐defined margins and irregular contrast in the arterial phase (a) and early drainage in the renal parenchymal phase (b). The right renal tumor is 26 mm in diameter and shows well‐defined margins without contrast‐enhancement in both arterial and renal parenchymal phases (c,d).

**Fig. 2:**
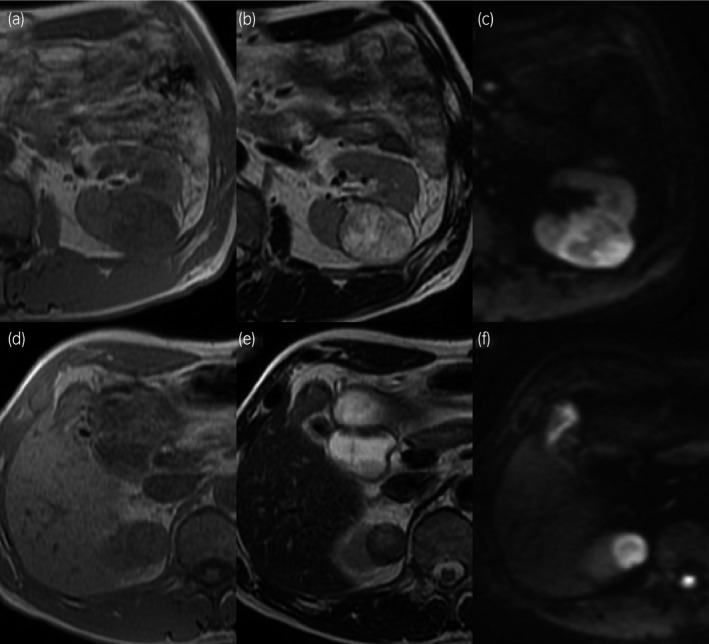
Abdominal MRI of bilateral renal tumors. The left renal tumor shows solid hypointensity on T1‐weighted imaging (a) and irregular hyperintensity on T2‐weighted imaging (b). The right renal tumor appears isointense on T1‐weighted imaging (d) and hypointense on T2‐weighted imaging (e). Both tumors show strong signals on DWI (left; c and right; f).

A 62‐year‐old man was introduced to our hospital after bilateral renal tumors were identified in a health screening. Contrast‐enhanced CT revealed a left renal tumor 42 mm in diameter, showing well‐defined margins, irregular contrast in the arterial phase and early drainage in the renal parenchymal phase (Fig. 1a,b). MRI revealed a solid, low‐intensity mass on T1‐weighted imaging, an irregular high‐intensity mass on T2‐weighted imaging, and strong signals on DWI (Fig. 2a–c). These findings led to an expectation of typical ccRCC in the left kidney. The right renal tumor was 26 mm in diameter and showed no contrast enhancement on CT (Fig. 1c,d). The mass was isointense on T1‐weighted imaging and hypointense on T2‐weighted imaging. DWI revealed strong signals (Fig. 2d–f). These findings suggested RCC with a non‐clear cell‐type histology in the right kidney. The patient was diagnosed clinically with cT1b ccRCC in the left kidney and cT1a non‐ccRCC in the right kidney, with no apparent metastases (N0M0 stage). Robot‐assisted partial nephrectomies were performed, first for the left renal tumor, then for the right renal tumor 3 months later.

**Fig. 3:**
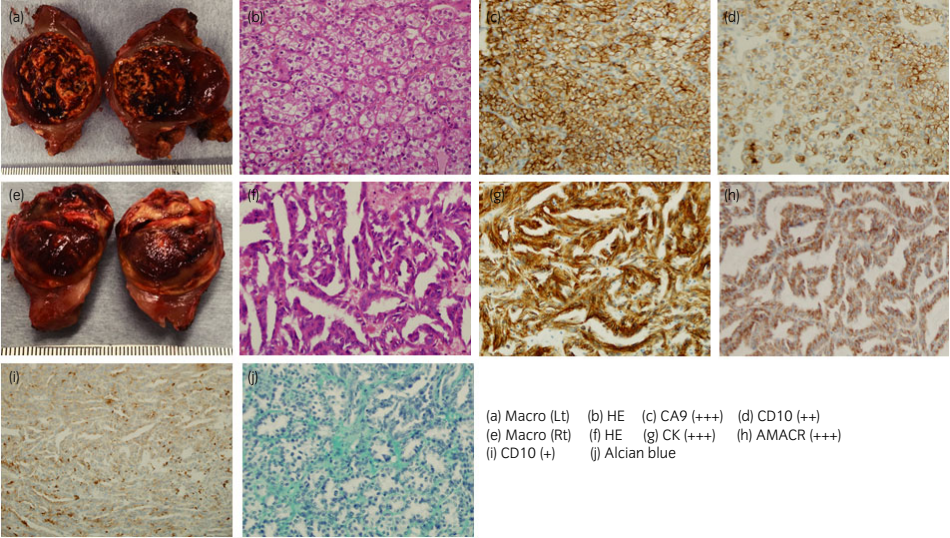
Macro‐ and microscopic findings of bilateral renal tumors. Both tumors were partially resected by robot‐associated laparoscopic surgery. The cross‐sectional surface of the left renal tumor is lobulated and heterogeneously yellow with bleeding and necrosis (a). The right renal tumor is a white and grayish‐white circular mass with internal hemorrhage and necrosis (e). HE staining of the left renal tumor shows an alveolar growth pattern with cells containing clear cytoplasm, and displays abundant vascular plexuses and sparse stroma (b). Moderate to strong immunostaining for CA9 (c) and CD10 (d) is seen on the cell membrane. HE staining of the right renal tumor shows a slim, tubular structure containing viscous liquid and spindle cells (f). Strong immunostaining for CK7 (g) and AMACR (h) is diffusely evident in cytoplasm. Weak focal positivity for CD10 is seen (i). Alcian blue staining identifies mucus in the stroma (j).

Macroscopic examination of the left renal tumor revealed a lobulated, heterogeneously yellow appearance with bleeding and necrosis. Macroscopic examination of the right renal tumor revealed a well‐defined, circular, solid mass lacking a pseudo‐capsule. The cut face was white and grayish‐white in color with internal hemorrhage and necrosis (Fig. 3a,e). Both tumors were examined histopathologically. Microscopic findings for the left renal tumor using HE staining included an alveolar growth pattern of cells with clear cytoplasm, abundant vascular plexuses, and sparse stroma. Immunohistochemical examination of the left renal tumor with anti‐CA9 and anti‐CD10 antibodies yielded strongly and moderately positive results, respectively, but results for CK7 were negative. These results identified the left renal tumor as ccRCC (Fig. 3b–d). Microscopic findings for the right renal tumor using HE staining showed cuboid cells consisting of tubular and papillary growth patterns with stromal mucin, and spindle cells were occasionally absorbed. Immunohistochemical examination of the right renal tumor with anti‐CK7 and anti‐AMACR antibodies yielded strongly positive results, while CD10 was weakly and focally positive. Mucus stained with Alcian blue was identified within the tumor stroma. These findings led to a diagnosis of MTSCC in the right kidney (Fig. 3f–j). The patient has shown no signs of recurrence as of 12 months postoperatively.

## Discussion

RCC is the most common malignant tumor arising from the kidney. Clear cell subtype is the most common histology, representing approximately 70% of RCCs.^3^

MTSCC is a rare kidney cancer and only limited information is available from the literature, but this pathology was recognized as a distinct entity in the 2004 World Health Organization tumor classification.^4^ MTSCC has been characterized by a wide age distribution, female predominance and generally low malignant potential and low risk of metastasis. Histological characterizations of MTSCC include a prominent spindle cell change, possibly related to the loop of Henle and presence of an admixture of low‐grade cuboidal cells in tubules and sheets of spindle cells, and variable amounts of mucinous stroma.^5^

The incidence of synchronously occurring bilateral RCCs is 1–5% among patients with RCC.^1^, ^2^, ^6^ Hereditary RCC often manifests as bilateral and multifocal RCCs, such as von Hippel–Lindau disease‐associated ccRCC.^7^ Klatte *et al*. reported that about 91% of 135 patients with synchronous bilateral RCCs in a multicenter experience had non‐hereditary bilateral RCCs. Bilateral ccRCC was the major histological subtype (73%), with bilateral papillary RCC as the second most common (16%), and bilateral chromophobe RCC as the third most common (4%).^6^ The remaining cases comprised bilateral RCC with different histologies (ccRCC with contralateral papillary RCC (6%) and papillary RCC with contralateral chromophobe RCC (1%)).^6^

Wang *et al*. reported the surgical management that staged bilateral retroperitoneoscopic partial nephrectomy was superior in renal functional preservation with equivalent oncological results compared with the sequencing radical nephrectomy. Partial nephrectomy and partial nephrectomy followed by radical nephrectomy in the patients with bilateral synchronous sporadic RCC.^8^ Focal ablation therapies, including cryoablation and radiofrequency ablation, have recently emerged as valid alternatives to nephron‐sparing surgery. The combination of partial nephrectomy and ablation therapy may be worth considering when bilateral partial nephrectomies prove difficult.^9^ Partial nephrectomy may facilitate the management of large, complex tumors, while ablation therapy may be suitable for small, well‐marginated tumors.^10^ The best possible prediction of expected histologies from preoperative diagnostic imaging is also important. Robot‐assisted partial nephrectomy was thus performed for the left renal tumor first in the present case, because of its larger, 42‐mm diameter compared to the 26‐mm right tumor. This staged surgery was tolerated well by our patient. Whether both sides should be operated on, whether surgery should be combined with ablation therapy, and which tumor should be treated first represent issues that should be considered based on imaging findings, the clinical course, and the background of the individual patient.

## Conclusion

We encountered a case of bilateral RCCs with synchronous onset. This appears to be the first report of different histologies (ccRCC and MTSCC) for synchronous RCCs.

## Conflict of interest

The authors declare no conflict of interest.

## Author contributions

RS and TK drafted the report and contributed to concept and design. SK, KA and DT contributed to data collection. TK, HF, SF and SA contributed to clinical work. IM and NK contributed to histopathological analyses. KI contributed to supervision and approved the final version of the manuscript. All authors read and approved the final manuscript.
